# Performance of triggers in detecting hospitalizations related to drug-induced respiratory disorders in older adults: A pilot cross-sectional study

**DOI:** 10.1016/j.clinsp.2024.100449

**Published:** 2024-07-27

**Authors:** Geovana Schiavo, Marcela Forgerini, Fabiana Rossi Varallo, Bruna Carolina Corrêa, Maisa Cabete Pereira Salvetti, Patrícia de Carvalho Mastroianni

**Affiliations:** a Department of Drugs and Medicines, Faculdade de Ciências Farmacêuticas da Universidade Estadual Paulista (UNESP), Araraquara, SP, Brazil; b Universidade de São Paulo (USP), Faculdade de Ciências Farmacêuticas de Ribeirão Preto, Ribeirão Preto, SP, Brazil; c Hospital Estadual Américo Brasiliense (HEAB), Faculdade de Medicina de Ribeirão Preto, Universidade de São Paulo (USP), Ribeirão Preto, SP, Brazil

**Keywords:** Aged, Chemically Induced disorders, Health care, Quality indicators, Safety management

## Abstract

**Background:**

There is no gold-standard trigger for detecting drug-induced respiratory disorders, a type of Adverse Drug Event (ADE) with high morbimortality, particularly in older people.

**Objective:**

To propose and evaluate the performance of triggers for detecting hospitalizations related to drug-induced respiratory disorders in older people.

**Methods:**

A pilot cross-sectional study was conducted with older people (age ≥ 60) admitted to a Brazilian hospital. Electronic chart documentation was screened using ICD-10 codes; *Global Trigger Tool* (GTT); and drugs potentially associated with respiratory disorders. A chart and medication review were conducted to perform the causality assessment using the instrument developed by the World Health Organization. The performance of triggers was evaluated by the Positive Predictive Value (PPV), with values ≥ 0.20 indicating good performance.

**Results:**

Among 221 older people, 72 were eligible. Potential drug-induced dyspnea and/or cough were detected in six older people (6/72), corresponding to a prevalence of 8.3 %. The overall PPV of the triggers was 0.14, with abrupt medication stop (PPV = 1.00), codeine (PPV = 1.00), captopril (PPV = 0.33), and carvedilol (PPV = 0.33) showing good performance. Two triggers were proposed for detecting therapeutic ineffectiveness associated with respiratory disorders: furosemide (PPV = 0.23) and prednisone (PPV = 0.20).

**Conclusion:**

The triggers enabled the identification that one in 12 hospitalizations was related to drug-induced respiratory. Although good performance was observed in the application of triggers, additional investigations are needed to assess the feasibility of incorporating them into clinical practice for the screening, detection, management, and reporting of these ADEs, which are considered to be underreported and difficult to detect.

## Highlights

• A prevalence of 8.3 % of drug-induced respiratory disorders was identified.
• Triggers detected hospitalizations related to drug-induced respiratory disorders.
• Four triggers showed good performance for detecting drug-induced respiratory disorder.
• Two triggers detected therapeutic ineffectiveness related to respiratory symptoms.

## Introduction

Considering the need for care and interventions within the scope of health, especially after the COVID-19 pandemic, there has been an exponential increase in studies evaluating respiratory disorders and their risk factors.^1^ However, there are still gaps in the literature regarding drug-induced respiratory disorders, which can be responsible for more than one respiratory disease and affect the entire respiratory system (e.g., airways and respiratory muscles).^2^

A drug-induced respiratory disorder is a type of Adverse Drug Event (ADE) with high morbidity and mortality^3^ and is characterized by the presence of nonspecific clinical signs and symptoms common to respiratory diseases (e.g., cough, dyspnea, and fever).^3^^,^^4^

Although it is known that older people, particularly those with a previous diagnosis of respiratory diseases or infections,^3^ who are receiving multiple drugs (polypharmacy),^5^ or using analgesics and antibiotics,^6^ are at greater risk of developing drug-induced respiratory disorders,^2^ there is a lack of studies designed to detect this type of ADEs and related hospitalizations. In addition, there is no consensus in the literature on whether drug-induced respiratory disorders have a low prevalence or if they are under-detected, with an estimated prevalence in older people around 7.5 %.^6^

Detecting drug-induced respiratory disorders is challenging and typically involves excluding other possible causes.^3^ In the absence of a gold-standard method, the application of triggers can be a valid strategy to detect and quantify the prevalence of drug-induced disorders, as they have shown good performance and utility in detecting ADEs and determining their preventability in health care settings.^7^ Furthermore, the easy application and reproducibility of triggers in different health services contribute to the planning and implementation of health interventions, thereby enhancing patient safety.^7^

In previous studies, triggers have demonstrated good performance in detecting ADEs, such as delirium and constipation in older people.^7^^,^^8^ Nonetheless, to the best of our knowledge, there is a lack of studies proposing, evaluating, or validating triggers for the detection of drug-induced respiratory disorders and related hospitalizations.^7^

Therefore, the aim of this pilot study was to propose and evaluate the performance of triggers in detecting drug-induced respiratory disorders related to hospitalizations in older people, as well as to estimate the prevalence of these ADEs in a Brazilian hospital.

## Methods

### Ethical aspects

This study was carried out by consulting electronic chart documentation with approval from the Research Ethics Committee of the São Paulo State University (UNESP) (CAAE 46938821.7.0000.5426).

### Study design and setting

A six-month pilot cross-sectional study with retrospective data collection was conducted to propose and evaluate the performance of triggers for detecting hospitalizations related to drug-induced respiratory diseases.

The study was conducted at Américo Brasiliense State Hospital (HEAB), located in the interior of the state of São Paulo, Brazil. The hospital has 84 beds and serves patients from the Regional Health Division III, which comprises 24 cities and approximately 989,971 inhabitants.

The reporting of this study was based on *Strengthening the Reporting of Observational Studies in Epidemiology* (STROBE) for cross-sectional studies.^9^

### Participants

Older people (age ≥ 60) admitted to general and surgical wards of the hospital between January and June 2021 were eligible. These two medical wards correspond to 38 beds, where patients are admitted regardless of their age or specific health conditions, for the diagnosis or management of morbidities and infections and the performance of health procedures (e.g., medical exams and surgeries).

The primary exclusion criteria were older people without signs and/or symptoms of respiratory disorders or those diagnosed with COVID-19, regardless of whether the diagnosis was made before or during hospitalization. Older people diagnosed with COVID-19 were not eligible due to the difficulty in distinguishing between signs and/or symptoms of the clinical condition and drug-induced respiratory disorders.

The secondary exclusion criteria consisted of older people who had not used drugs before hospital admission.

**Fig. 1:**
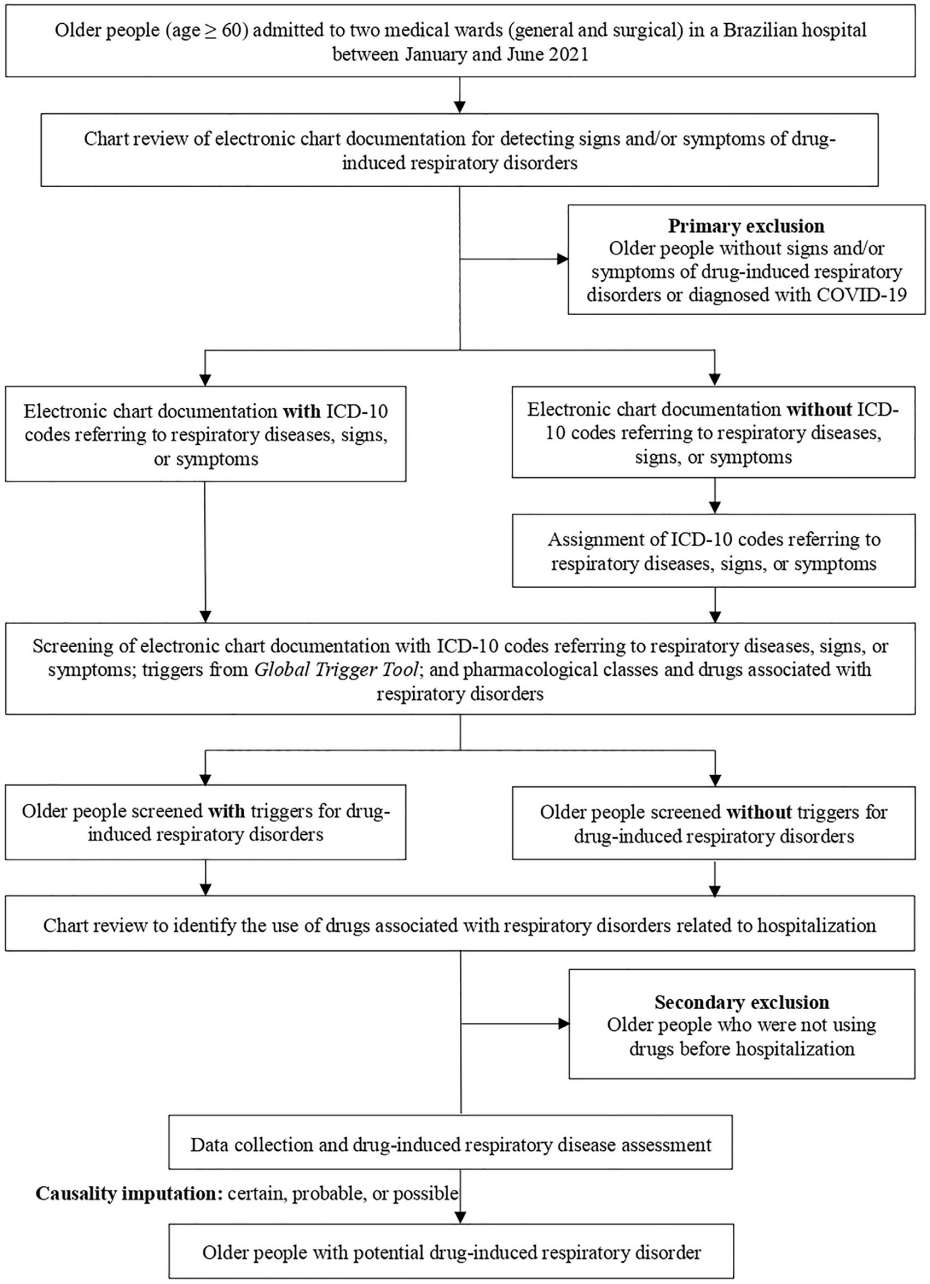
Screening of triggers and data collection and medication review in electronic chart documentation of older people admitted to two medical wards of Américo Brasiliense Hospital, January to June 2021. ICD-10, International Code of Diseases-10^th^ Revision.

Hospitalizations and readmissions from the two wards were evaluated during the study period to obtain a robust sample. The flowchart of the participant recruitment process is shown in Fig. 1.

### Screening for drug-induced respiratory disorders

The triggers were screened in the electronic chart documentation of the multidisciplinary team (physicians, pharmacists, nurses, and physical therapists) and in therapeutic prescriptions.

Given the absence of specific triggers for detecting hospitalizations related to drug-induced respiratory disorders, the following screening strategies were adopted:

(i) International Code of Diseases 10^th^ Revision (ICD-10) codes*:* screening was carried out by identifying at least one of the codes referring to respiratory diseases, signs, or symptoms (Supplementary Material 1). In view of the routine and time constraints of the medical team,^10^ not all ICD-10 codes referring to diseases, signs, or symptoms were assigned in electronic chart documentation. To ensure the screening of these triggers, a researcher (G. S) assigned relevant ICD-10 codes for older people who had a diagnosis of respiratory diseases (e.g., asthma) or signs and/or symptoms of respiratory disorders (e.g., dyspnea).
(ii) Triggers from *Global Trigger Tool* (GTT):^11^ The potential triggers present in the GTT that suggest the presence of a potential drug-induced respiratory disease are listed in Supplementary Material 2.
(iii) Pharmacological classes and drugs associated with respiratory disorders: screening consisted of identifying drugs that are potentially associated with respiratory disorders according to scientific evidence.^12^^,^^13^ Their screening in the electronic chart documentation of older people with signs and/or symptoms of respiratory disorders suggests the presence of ADEs. Pharmacological classes and drugs used during the hospitalization were not considered as triggers (Supplementary Material 3).

### Data collection and variables

Data collection was conducted by consulting the electronic chart documentation using a review form previously designed by the researchers (Supplementary Material 4). The following variables were recorded:

Sociodemographic: Sex; age (young older people [age ≥60–79] and long-lived older people [age ≥ 80]; and self-declared color (white, brown [*“Pardo”* in Brazilian Portuguese], and black).

Clinical: Body Mass Index (BMI) (underweight [< 18 kg/m^2^], normal [≥ 18–≤ 24 kg/m^2^], overweight [≥ 25–≤ 29.9 kg/m^2^], and obesity [≥ 30 kg/m^2^]); outcome (death or hospital discharge); days of hospitalization; and diseases. The presence of multimorbidity, defined by the World Health Organization as the presence of two or more morbidities, was assessed.^14^

Lifestyle: Smoking habits and alcohol intake.

### Drug-induced respiratory disorders assessment

Causality assessment of ADEs

An ADE was defined as any harmful event occurring during the use of drug therapy, regardless of dose.^15^ A drug-induced respiratory disorder was defined as an ADE with signs and symptoms affecting the entire respiratory system (e.g., pulmonary parenchyma and airways).^16^

Since this study aimed to identify hospitalizations related to drug-induced respiratory disorders, only drugs used before hospitalization were assessed. Additionally, during hospitalization, ADEs were managed, as clinical pharmacists and physicians conducted the medication review and assessed the risks *versus* benefits of pharmacotherapy.

Causality assessment was performed for all hospitalizations and readmissions using the instrument developed by the World Health Organization.^17^ The assessments were carried out by one researcher (Author 1) and supported by discussions with two other researchers (M. F and F. R. V). The choice of this instrument is justified by its greater consistency in imputing ADEs in hospitals.^18^

Factors such as the temporal relationship between drug use and potential respiratory disorder, scientific evidence, pharmacological plausibility, and confounding variables (e.g., diagnosis of respiratory diseases and smoking habit) were considered. Causality assessments with the imputation as certain, probable, and possible were considered as potential drug-induced respiratory disorders.

Preventability and severity of ADEs

This study assessed whether drug-induced respiratory disorders could have been avoided, based on the following definitions:^19^

– Non-preventable: adverse drug reactions that cannot be prevented as they result from the intrinsic properties of the drug itself.
– Preventable: medication errors that might lead to patient injury.

For preventable drug-induced respiratory disorders, severity was classified according to the medication error classification defined by the *National Coordinating Council for Medication Error Reporting and Prevention*.^20^ Only categories describing patient harm were considered: E (an error occurred that might have contributed to or resulted in temporary harm to the patient and required intervention) and F (an error occurred that might have contributed to or resulted in temporary harm to the patient and required initial or prolonged hospitalization).

#### Therapeutic ineffectiveness

The therapeutic ineffectiveness of certain drugs may induce or exacerbate respiratory signs, symptoms, and disorders. Ineffectiveness was defined as the absence or reduction of the expected therapeutic effectiveness of the drug under the prescribed or indicated conditions of use.^21^ Medical reports were evaluated to identify potential cases of therapeutic ineffectiveness associated with signs and/or symptoms of respiratory disorders.

### Statistical methods

Sociodemographic, clinical, pharmacotherapeutic, and lifestyle variables were presented descriptively using absolute and relative frequency. For continuous variables, mean and standard deviation were reported.

To assess the statistical difference between the groups (older people with and without drug-induced respiratory disorder), Fisher's exact test for qualitative variables or the *t*-Student for quantitative variables was used.

### Performance of the triggers

To evaluate the performance of the triggers in detecting drug-induced respiratory disorders, the Positive Predictive Value (PPV) was calculated according to the following equation: $\mathrm{PPV}=\frac{\text{Number}\;\text{of}\;\text{older}\;\text{people}\;\text{with}\;\text{potential}\;\text{drug}-\text{induced}\;\text{respiratory}\;\text{disorders}\;\text{detected}\;\text{by}\;\text{the}\;\text{triggers}}{\text{Number}\;\text{of}\;\text{older}\;\text{people}\;\text{with}\;\text{signs}\frac{\text{and}}{\text{or}}\text{symptoms}\;\text{of}\;\text{drug}-\text{induced}\;\text{respiratory}\;\text{disordersscreened}\;\text{by}\;\text{the}\;\text{triggers}}$

PPV was performed only for triggers that detected potential drug-induced respiratory disorders, values ≥ 0.20 were considered a good performance.^22^

### Data accessibility

The supplementary material is available at Open Science Framework (doi:10.17605/OSF.IO/EY6MX).

## Results

### Characteristics of older people

**Fig. 2:**
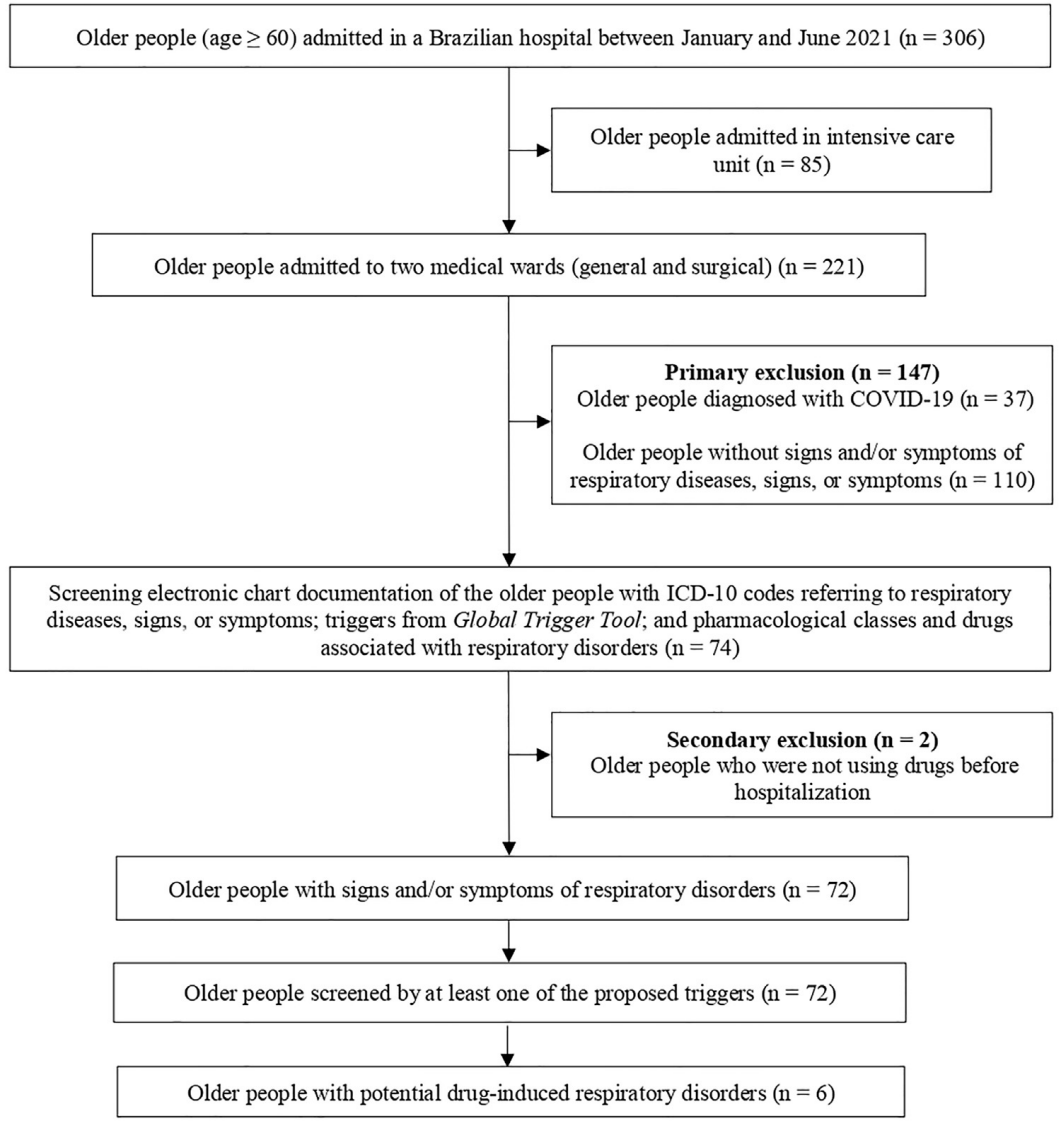
Flowchart of detection of potential drug-induced respiratory disorders in older people admitted to two medical wards of the Américo Brasiliense Hospital, São Paulo, January to June 2021. ICD-10, International Code of Diseases-10^th^ Revision.

Between January and June 2021, 306 older people were admitted to the hospital in the study (346 hospitalizations). In the selected general and surgical wards, 221 older people were admitted (251 hospitalizations) and 72 older people were eligible (81 hospitalizations) (Fig. 2).

The greater number of hospitalizations is explained by the fact that an older person may have been admitted to the selected wards more than once during the data collection period.

Among the 72 older people who met the eligibility criteria, most were male (*n* = 37), with a mean age of 72 years (SD ± 8), and a mean length of hospital stay of 11 days (SD ± 10). The most frequent diseases were high blood pressure (*n* = 51) and diabetes *mellitus* (*n* = 29). Thirty older people had a diagnosis of respiratory diseases, four had a history of previous respiratory infections, and three were diagnosed with respiratory infections during hospitalization. Most of the participants were abstainers (*n* = 49) and nonsmokers (*n* = 58) (Table 1).

**Table 1:** Baseline characteristics of older people admitted to two medical wards of the Américo Brasiliense Hospital (*n* = 72) from January to June 2021, Brazil.

**Variable**	**Older people without potential drug-induced respiratory (***n* = **66)**	**Older people with potential drug-induced respiratory (***n* = **6)**	**p-value**
Sex (men)	35	2	0.423
Days hospitalized (mean ± SD)^a^	10 ± 9	14 ± 14	0.218
Outcome (death)	11	1	1.000
**Age (years)**			0.598
Young older people (age ≥ 60‒79)^b^	53	4	
Long-lived older people (age ≥ 80)	13	2	
Self-declared color			1.000
White	52	6	
Brown ("*Pardo*" in Brazilian Portuguese)	8	0	
Black	6	0	
**Body mass index (kg/m^2^)**			0.403
Normal (≥ 18–≤ 24)	16	2	
Underweight (< 18)	8	1	
Overweight (≥ 25–≤ 29.9)	26	0	
Obesity I, II, and III (> 30)	14	3	
Disease			
High blood pressure	46	5	0.431
Respiratory disease (diagnosed before to hospital admission)	26	4	0.193
Diabetes *mellitus*	25	4	0.212
Dyslipidemia	23	4	0.136
Multimorbidity (presence of two or more morbidities)	41	5	0.030
**Alcohol intake**	14	0	0.328
**Smoking habit**	6	2	0.200

95 % CI, Confidence Interval 95 %; OR, Odds Ratio; SD, Standard Deviation.

a p-value obtained in the t-Student test. The p-value of other variables were obtained in the Fisher's exact test (the number of participants in one or more categories is less than five).

### Screening and performance of the proposed triggers

The 72 eligible older people had at least one trigger identified, with an average of six triggers screened (SD ± 1). The most screened triggers were the ICD-10 codes referring to respiratory diseases, signs, or symptoms (*n* = 214), mainly cough (*n* = 52) and dyspnea (*n* = 48), followed by pharmacological classes and drugs associated with respiratory disorders (*n* = 130).

The most identified pharmacological classes during the screening were beta-blockers (*n* = 38) and calcium channel antagonists (*n* = 20), while the drugs were aspirin (*n* = 21) and hydrochlorothiazide (*n* = 11). The triggers from GTT were screened 18 times, especially the trigger “transfer to a higher level of care” (*n* = 14) (Supplementary Material 5).

The total number of screened triggers is greater than the number of hospitalizations, as a case of potential drug-induced respiratory disorder could be screened by more than one trigger (Fig. 2).

The overall PPV of the triggers for detecting drug-induced respiratory was 0.14. The triggers that presented good performance for detecting drug-induced respiratory disorders (PPV ≥ 0.20) were abrupt medication stop (PPV = 1.00) and the use of codeine (PPV = 1.00), carvedilol (PPV = 0.33), and captopril (PPV = 0.33) (Table 2).

**Table 2:** Positive predictive value of triggers that detect potential drug-induced respiratory disorders related to hospital admissions and therapeutic ineffectiveness in older people admitted to two medical wards of the Américo Brasiliense Hospital (*n* = 72) from January to June 2021, Brazil.

**Trigger**	**Number of times screened in electronic chart documentation**	**Number of times detected potential ADE or therapeutic ineffectiveness**	**PVV**
**Potential drug-induced respiratory disorder**			
**International Code of Diseases-10^th^ Revision codes (ICD-10)**			
Dyspnea	48	7	0.14
Cough	52	3	0.06
Trigger adapted from *Global Trigger Tool*			
Abrupt medication stop	1	1	1.00
**Drug (pharmacological class)**			
atenolol (beta-blocker)	12	1	0.08
captopril (angiotensin converting enzyme inhibitor)	3	1	0.33
carvedilol (beta-blocker)	6	2	0.33
clonazepam (benzodiazepine)	11	2	0.18
codeine (opioid)	1	1	1.00
enalapril (angiotensin converting enzyme inhibitor)	11	1	0.09
**Therapeutic ineffectiveness**			
budesonide/formoterol (glucocorticoid/beta-2 adrenergic agonist)	11	1	0.09
furosemide (loop diuretic)	17	4	0.23
prednisone (glucocorticoid)	5	1	0.20
**Total**	**178**	**25**	**0.14**

ADE, Adverse Drug Event; PVV, Positive Predictive Value.

### Causality assessment and prevalence of drug-induced respiratory disorder

A causality assessment was performed for 72 older people (81 hospitalizations), evaluating 319 drugs associated with potential respiratory disorders.

Regarding drug-induced respiratory disorders, six older people had causality assessments with the imputation as possible for eight drugs associated with respiratory disorders (Table 3), corresponding to a prevalence of 8.3 % (6/72). Cough and/or dyspnea were the potential drug-induced respiratory disorders identified in six older people, contributing to or causing hospital admission. Drugs associated with dyspnea were clonazepam (*n* = 2), atenolol (*n* = 1), carvedilol (*n* = 1), codeine (*n* = 1), atenolol (*n* = 1), and trazodone (*n* = 1), while drugs associated with cough were captopril (*n* = 1), carvedilol (*n* = 1), and enalapril (*n* = 1) (Supplementary Material 6).

**Table 3:** Description of the clinical history of the six older people with a potential drug-induced respiratory disorders related to hospital admissions.

**Clinical history**	**Drugs in use before the hospitalization**	**Hospital admission**	**Drug-induced respiratory disorder assessment**	**Triggers screened**	**Causality assessment (medication error)**
**Patient:** 63-year-old woman.	aminophylline 200 mg (1-1-1); amitriptyline 25 mg (0-0-1); amlodipine (25 mg, 1-0-1); azithromycin 500 mg (Monday, Wednesday, and Friday/0-0-1); clonazepam 2 mg (0-0-1); codeine 30 mg (1-1-1); formoterol and budesonide 12 mcg and 400 mcg (1-0-1); indacaterol 150 mcg/day; salbutamol 100 mcg (1-1-1-1-1-1); sertraline 150 mg/day; and tiotropium 2.5 mcg (2-0-2).	Hospitalized for eight days due to an exacerbation of dyspnea and cough.	Upon hospital admission, the use of azithromycin and codeine was discontinued, and the use of morphine and dexamethasone was initiated. An improvement in dyspnea was observed, which might be attributed to the suspension of codeine and the use of morphine and dexamethasone.	**ICD-10 Codes:** J441, J448, R05, J80.	Possible for codeine induced dyspnea (category F).
**Comorbidities:** chronic obstructive pulmonary disease, depressive disorder, diabetes *mellitus,* fibromyalgia, high blood pressure, and osteoporosis. History of exposure to a wood stove for 12 years.				**Drug (pharmacological class):** clonazepam (benzodiazepine), amlodipine (calcium channel blocker), and codeine (opioid).	
**Life habits:** abstainer and smoker.					
**Patient:** 68-year-old woman.	captopril 25 mg (1-0-1); formoterol and budesonide 12 mcg and 400 mcg (1-0-1); and metformin 850 mg (1-1-1).	Hospitalized for 10 days due for an investigation of lung neoplasm and chronic cough.	The enalapril dosage was reduced and the cough improves.	**ICD-10 Codes:** C493, R05, R042, R91.	Possible for enalapril induced cough (category E).
**Comorbidities:** chronic obstructive pulmonary disease, diabetes *mellitus,* high blood pressure, and lung neoplasm.				**Drug (pharmacological class):** captopril (angiotensin converting enzyme inhibitor).	
**Life habits:** abstainer and nonsmoker.					
**Patient:** 61-year-old woman.	atenolol 25 mg (2-0-2); clonazepam 2 mg (0-0-1); formoterol and budesonide 12 mcg and 400 mcg (1-0-1); hydrochlorothiazide 25 mg/day; losartan 50 mg (1-0-1); salbutamol 100 mcg (if necessary); sertraline 50 mg (2-0-0); atorvastatin 50 mg (0-0-1); and tramadol 50 mg (1-0-0).	Hospitalized for two days due an exacerbation of chronic obstructive pulmonary disease, dyspnea and cough.	Upon hospital admission, the use of clonazepam and atenolol was discontinued, and it was observed an improvement in respiratory symptoms.	**ICD-10 Codes:** J441, J98, R05, R60.	Possible for atenolol and clonazepam induced dyspnea (category F).
**Comorbidities:** anxiety disorder, chronic obstructive pulmonary disease, and high blood pressure.				**Drug (pharmacological class):** atenolol (beta-blocker), and clonazepam (benzodiazepine)	
**Life habits:** abstainer and smoker.				**Trigger from *Global Trigger tool*:** Abrupt medication stop	
**Patient:** 80-year-old man.	aspirin 100 mg (0-1-0); enalapril 10 mg (1-0-1); metformin 500 mg (1-1-1); phenobarbital 100 mg (0-0-1); quetiapine 25 mg (0-0-1); and simvastatin 40 mg (0-0-1).	Hospitalized for 42 days due an exacerbation of cough and bronchopneumonia.	Upon hospital admission, captopril was discontinued, and amoxicillin was prescribed, with improvement in respiratory signs.	**ICD-10 Codes:** J180, R05, R60.	Possible for captopril induced cough.
**Comorbidities:** diabetes *mellitus,* dyslipidemia, and high blood pressure.				**Drug (pharmacological class):** enalapril (angiotensin converting enzyme inhibitor),and phenobarbital (barbiturates).	
**Drugs in use before the hospitalization:**					
**Life habits:** abstainer and nonsmoker.					
**Patient:** 71-year-old man.	amiodarone 200 mg/day; carvedilol 5 mg (1-0-1); furosemide 40 mg (1-0-0); losartan 50 mg (1-0-0); metformin 500 mg (1-1-1); omeprazole 20 mg (1-0-0); simvastatin 40 mg (0-0-1); and warfarin 2.5 mg/day (Monday to Friday).	Hospitalized for 13 days due to an exacerbation of cough and dyspnea.	Upon hospital admission, carvedilol was discontinued and meropenem was prescribed, with improvement in respiratory signs.	**ICD-10 Codes:** J189, R05, R60, J80.	Possible for carvedilol induced cough.
**Comorbidities:** atrial fibrillation, diabetes *mellitus,* and high blood pressure.				**Drug (pharmacological class):** amiodarone (antiarrhythmic), and carvedilol (beta-blocker).	
**Life habits:** abstainer and nonsmoker.					
**Patient:** 84-year-old woman.	baclofen 5 mg (1-0-1); dipyrone 1000 mg (1-1-1-1); scopolamine 20 mg (1-1-1); metoprolol 50 mg (2-0-2); thiamazole 15 mg/day; and trazodone 50 mg/day.	Hospitalized for eight days for palliative care and management of bronchopneumonia and dyspnea.	Upon hospital admission, the use of clonazepam and trazodone was discontinued. The patient showed improvement in respiratory signs, but the patient evolves to death.	**ICD-10 Codes:** J180, J80, R60.	Possible for clonazepam and trazodone induced dyspnea.
**Comorbidities:** atrial fibrillation, cardiomyopathy, depressive disorder, dyslipidemia, epilepsy, high blood pressure, and sleep apnea.				**Drug (pharmacological class):** metoprolol (beta-blocker).	
**Life habits:** abstainer and nonsmoker.					

Severity was assigned to participants with respiratory disorders caused by medication errors.

ICD-10 Codes, International Code of Diseases-10^th^ Revision codes (ICD-10); C493, Malignant neoplasm of connective and soft tissue of thorax; J180, Bronchopneumonia, unspecified organism; J189, Pneumonia, unspecified organism; J441, Chronic obstructive pulmonary disease with (acute) exacerbation; J448, Other specified forms of chronic obstructive pulmonary disease; J80, Acute respiratory distress syndrome; J98, Other respiratory disorders (tachypnea); R042, Hemoptysis; R05, Cough. R60: Dyspnea; R91, Abnormal findings on diagnostic imaging of lung.

Causality assessment as probable or certain for drug-induced respiratory disorders was not possible for these six older people. Four older people had a diagnosis of respiratory diseases or a clinical condition justifying respiratory signs and/or symptoms, and two did not have reports of withdrawal and rechallenge of the drug associated with the potential respiratory disorder – criteria required to impute an ADE as probable or certain (Supplementary Material 6).

The assessment of the degree of causality between the occurrence of potential respiratory disorders and the use of pharmacotherapy is described in Supplementary Material 6.

### Preventability and severity of ADEs

Potential drug-induced respiratory disorders could have been prevented in three older people, as the patients had a diagnosis of chronic obstructive pulmonary disease and had experience with the use of drugs associated with exacerbation of respiratory disorders before hospital admission (i.e., codeine, enalapril, atenolol, and clonazepam).

This ADE was responsible for the hospital admission of two older people (category F), and in one case, the disorder was identified during the hospital admission for the investigation of neoplasm (category E).

### Therapeutic ineffectiveness

None of the six older people with potential drug-induced respiratory disorders presented therapeutic ineffectiveness (6/72). Among the other 66 eligible older people (66/72), six had potential cases of therapeutic ineffectiveness, as reported by the physician. Four had pulmonary edema due to an inappropriate dosage of furosemide (underuse); one had dyspnea due to the use of lower doses of formoterol and budesonide; and one presented a cough due to the use of a lower dose of prednisone.

For the detection of therapeutic ineffectiveness induced by an inappropriate dosage of the drug, it was possible to propose two drugs as triggers: furosemide for detecting pulmonary edema (PPV = 0.23) and prednisone for detecting cough (PPV = 0.20) (Table 2).

## Discussion

In this pilot study, the proposed triggers allowed the detection of drug-induced respiratory disorders related to hospitalizations in older people. The triggers “abrupt medication stop” and the “use of codeine, captopril, and carvedilol” demonstrated good performance and enabled the identification of medication errors with harm (categories E and F). Additionally, two drugs used as triggers showed good performance in detecting therapeutic ineffectiveness induced by inappropriate dosages (furosemide and prednisone use).

Although the ICD-10 codes were the most frequently screened triggers in electronic chart documentation and detected all cases of drug-induced respiratory disorders, they demonstrate poor performance and a high rate of false positives. This might be explained by the ICD-10 codes referring mainly to the diagnosis of pre-existing respiratory diseases (e.g., asthma) and associated signs and/or symptoms (e.g., dyspnea).

One of the novelties of this study was the proposal to use pharmacological classes and drugs potentially associated with respiratory disorders as triggers.^12^^,^^13^ Considering their good performance, easy applicability, and the limited time available to health professionals in clinical practice,^23^ these triggers might facilitate the detection and management of ADEs during the care process and reduce health problems. However, there are some caveats to be considered. While some triggers are more practical and specific for the detection of certain types of ADEs (e.g., naloxone for opioid intoxication), others are general and require additional time to detect ADEs (e.g., administration of antihistamines).^22^^,^^24^

Among the triggers from the GTT, “abrupt medication stop” demonstrated good performance in detecting dyspnea induced by atenolol. However, this trigger was detected only once, and its applicability in clinical practice may be limited since it requires a thorough review of all patient electronic chart documentation and potential causes of abrupt medication stops. Despite being widely used,^25^ the triggers from the GTT demonstrated better performance in detecting other types of ADEs, mainly constipation and hypotension.^26^ Furthermore, it is not possible to compare the present findings with the literature, as to the best of our knowledge, there are no reports of the GTT's performance in detecting drug-induced respiratory disorders in older people.

The authors proposed two triggers for detecting therapeutic ineffectiveness. Underuse of furosemide was associated with exacerbation of heart failure resulting in pulmonary edema, while underuse of prednisone, budesonide, and formoterol was responsible for exacerbation of respiratory diseases.^27^ These triggers, like the pharmacological classes and drugs, are practical and specific and can be useful in clinical practice for detecting therapeutic ineffectiveness.

It is noteworthy that this study was conducted during the COVID-19 pandemic, a period that required heightened attention to respiratory disorders. There was encouragement from pharmaceutical industries and regulatory agencies to report possible ADEs associated with vaccines.^28^ Despite a significant increase in ADEs notifications, especially in Brazil,^28^ no increase in reports of drug-induced respiratory disorders was observed. The most frequent reports were related to hydroxychloroquine, mainly due to its irrational use for COVID-19 prophylaxis, and vaccines against COVID-19.^28^

In clinical practice, the application of triggers during medication review can allow the detection and monitoring of ADEs and help distinguish between signs and/or symptoms of respiratory disease exacerbation from drug-induced respiratory disorders.^29^ Consequently, medication review associated with the use of triggers enables the optimization of drug therapy^23^ and contributes to the design of a care plan for the prevention of iatrogenic cascade and the enhance of patient safety.^30^

Nevertheless, some aspects should be considered. Triggers need to be selected according to the prevalence of ADEs, performance (PPV), target population, health service, and available resources (e.g., data register in electronic chart documentation).^23^ In addition, the routine of health professionals may impair the screening of ADEs with triggers.^23^ The number of staff, time constraints, and non-automation of the process are relevant factors when selecting triggers to be incorporated into practice.

Moreover, the performance of triggers may be influenced by the prevalence of ADEs, the level of health service, differences in diagnostic, and therapeutic practices among health professionals,^31^ and institutional protocols.^32^ These factors may explain the absence of a consensus in the literature about the PPV considered as the gold standard, with good performance PVV ranging from 0.05 to 0.50.^7^ In this study, PPV ≥ 0.20 was adopted as good performance due to the low prevalence of drug-induced respiratory disorders, their difficulty in detection, and their similarity with respiratory diseases. However, PPV ≥ 0.20 for other types of ADEs, such as falls and fractures induced by nervous system depressants, may be considered poor performance since these types of ADEs are prevalent in clinical practice.^33^

Through the application of triggers, was estimating a prevalence of 8.3 % of older people admitted to a hospital with potential drug-induced respiratory disorders. This prevalence is similar to that reported in a study conducted by Woo and colleagues (2020), who identified a 7.5 % prevalence of drug-induced respiratory disorders among adverse drug reaction reports in a Korean database.^9^ The prevalence identified in the present study might be underestimated due to the small sample size and possible under-detection of drug-induced respiratory disorders, given the diagnosis of previous respiratory diseases in about half of the older people and missing data on the use of certain drugs influenced the imputation of ADEs.

This study has strengths and limitations. As strengths, it is noteworthy that this study is a groundbreaking proposal, as there is no previous study that evaluated the performance of triggers for detecting hospitalizations related to drug-induced respiratory disorders. Additionally, this study strengthens pharmacovigilance signals, which are still a bottleneck in Brazil, since the detection of drug-induced respiratory disorders was only possible through screening with the proposed triggers, as ADEs were not reported in electronic chart documentation.

As limitations, considering this was a pilot study, the sample size and analysis period should be highlighted. A total of 72 older people admitted to two wards in a Brazilian hospital were assessed over a six-month period and seasonality was not considered. Consequently, the findings may not reflect the reality of other hospitals or health facilities, limiting the inference of the data. To mitigate these possible biases, all hospitalizations during the study period were evaluated. In addition, another limitation was missing data, a barrier commonly found in studies that use secondary sources of information (electronic chart documentation).^7^

Considering the aforementioned aspects, future research is needed to evaluate the performance and usefulness of triggers for detecting hospitalizations related to drug-induced respiratory disorders in older people. Furthermore, improving the reporting in electronic chart documentation is required, and applying these triggers with prospective data collection could be a viable strategy for detecting and preventing this type of ADE.

## Conclusion

In this pilot study, one in 12 hospitalizations was related to cough and/or dyspnea induced by atenolol, captopril, carvedilol, clonazepam, codeine, enalapril, and trazodone. Half of the potential drug-induced respiratory diseases could have been avoided, as the older people had previous pulmonary impairment. Among the proposed triggers, “abrupt medication stop” and “the use of codeine, captopril, and carvedilol” showed good performance in detecting hospitalizations related to drug-induced respiratory disorders, while furosemide and prednisone use were effective in detecting therapeutic ineffectiveness.

The results obtained in this study are promising, and further studies are required to assess the performance and usefulness of incorporating these triggers into clinical practice for screening, detection, management, and reporting of this type of ADE, which is generally underreported and difficult to detect. This could help reduce drug therapy-related problems and enhance patient safety.

## Authors’ contributions

G.S. participated in the conception, methodology, patients screening, data collection, data curation, statistical analysis, data discussion and writing-original draft, writing review, and editing. M.F. participated in project administration, conception, methodology, data curation, statistical analysis, data discussion, and writing an original draft. F.R.V. participated in the conception, participated in methodology, data curation, data discussion, and revised the manuscript. B.C.C. participated in the conception, methodology, data discussion, revised manuscript, and project administration. M.C.P.S. participated in the conception, methodology, data discussion, revised manuscript, and project administration. P.C.M. participated in project administration, funding acquisition, supervision, validation, conception, methodology, statistical analysis, data discussion, and revised the manuscript. All authors read and approved the final version of the manuscript.

## Declaration of competing interest

The authors declare that they have no known competing financial interests or personal relationships that could have appeared to influence the work reported in this paper.

## Appendix Supplementary materials

Image, application 1

## Footnotes

Supplementary material associated with this article can be found, in the online version, at doi:10.1016/j.clinsp.2024.100449.
