# Supplementary material for: Performance of triggers in detecting hospitalizations related to drug-induced respiratory disorders in older adults: A pilot cross-sectional study
Source: Clinics (Sao Paulo). 2024 Jul 27;79:100449. doi: 10.1016/j.clinsp.2024.100449 (PMC11332799; doi:10.1016/j.clinsp.2024.100449)
Supplement: Supplementary file 1 [file mmc1.docx]

**Data availability**

The supplementary material associated with this article can be found in Open Science Framework (doi:10.17605/OSF.IO/EY6MX).
